# Escitalopram attenuates β-amyloid-induced tau hyperphosphorylation in primary hippocampal neurons through the 5-HT_1A_ receptor mediated Akt/GSK-3β pathway

**DOI:** 10.18632/oncotarget.7798

**Published:** 2016-02-29

**Authors:** Yan-Juan Wang, Qing-Guo Ren, Wei-Gang Gong, Di Wu, Xiang Tang, Xiao-Li Li, Fang-Fang Wu, Feng Bai, Lin Xu, Zhi-Jun Zhang

**Affiliations:** ^1^ Department of Neurology, ZhongDa Hospital, Neuropsychiatric Institute, Medical School of Southeast University, Nanjing, China; ^2^ Key Laboratory of Animal Models and Human Disease Mechanisms, Chinese Academy of Sciences, Kunming Institute of Zoology, Kunming, China; ^3^ Center of Schizophrenia, Beijing Institute for Brain Disorders, Beijing, China

**Keywords:** Alzheimer's disease, tau protein, escitalopram, Akt/GSK-3β pathway, 5-HT1A receptor, Gerotarget

## Abstract

Tau hyperphosphorylation is an important pathological feature of Alzheimer's disease (AD). To investigate whether escitalopram could inhibit amyloid-β (Aβ)-induced tau hyperphosphorylation and the underlying mechanisms, we treated the rat primary hippocampal neurons with Aβ_1-42_ and examined the effect of escitalopram on tau hyperphosphorylation. Results showed that escitalopram decreased Aβ_1–42_-induced tau hyperphosphorylation. In addition, escitalopram activated the Akt/GSK-3β pathway, and the PI3K inhibitor LY294002 blocked the attenuation of tau hyperphosphorylation induced by escitalopram. Moreover, the 5-HT_1A_ receptor agonist 8-OH-DPAT also activated the Akt/GSK-3β pathway and decreased Aβ_1-42_-induced tau hyperphosphorylation. Furthermore, the 5-HT_1A_ receptor antagonist WAY-100635 blocked the activation of Akt/GSK-3β pathway and the attenuation of tau hyperphosphorylation induced by escitalopram. Finally, escitalopram improved Aβ_1–42_ induced impairment of neurite outgrowth and spine density, and reversed Aβ_1–42_ induced reduction of synaptic proteins. Our results demonstrated that escitalopram attenuated Aβ_1–42_-induced tau hyperphosphorylation in primary hippocampal neurons through the 5-HT_1A_ receptor mediated Akt/GSK-3β pathway.

## INTRODUCTION

Alzheimer's disease (AD) is the most common cause of dementia in elderly people. Neurofibrillary tangles, composed of abnormally hyperphosphorylated tau, are key lesions of AD [1]. Abnormal hyperphosphorylation of tau converts it from a microtubule assembly-promoting to a microtubule-disrupting protein, leading to the destabilization of microtubules, the impairment of axonal transport, the dysfunction of hippocampal synaptic plasticity, and eventually the neuronal death [2]. Animal studies have consistently shown that the abnormal hyperphosphorylation of tau causes cognitive impairment [3, 4]. Therefore, proper manipulation of tau abnormal hyperphosphorylation could be promising for arresting AD neurodegeneration.

The involvement of the serotonin (5-HT) system in higher cognitive processes, such as learning and memory, has been widely described over the years and resurfaced as a new target for AD treatment. Postmortem and imaging studies demonstrated that the reduction of 5-HT and 5-HT_1A_ receptor (5-HT_1A_R) in the hippocampus is correlated with cognitive decline in AD patients [5, 6]. Selective serotonin reuptake inhibitors (SSRIs), a well-known class of antidepressants, act by selectively inhibiting the reuptake of 5-HT and subsequently increase the amount of serotonin available to bind critically to 5-HT_1A_R. SSRIs have been proved effective in hindering the progression of the AD and improving patients' performance [7-9]. Preclinical studies have also demonstrated a favorable cognitive-improving effect of SSRIs [10, 11]. SSRIs are also reported to increase neurotrophic factors including brain-derived neurotrophic factor (BDNF), promote neurogenesis in the hippocampus and reduce levels of toxic Amyloid-β (Aβ) [12, 13]. Interestingly, our previous study showed that escitalopram, one of the SSRIs, attenuated forskolin-induced tau hyperphosphorylation in human embryonic kidney cells that stably express human longest tau isoform tau441 (HEK293/tau441 cells) [14]. However, the mechanism has not been fully investigated.

At present, it is considered that glycogen synthase kinase-3β (GSK-3β) is a major tau kinase involved in tau hyperphosphorylation. GSK-3β activity is abnormally upregulated due to the inactivation of its upstream PI3K/Akt pathway in AD patients [15]. An activation of cortical GSK-3β has been found in 5-HT deficient mice [16, 17]. On the other hand, *in vivo* studies demonstrated that SSRI fluoxetine inactivated GSK-3β [18] and prevented the stress-induced inhibition of PI3K/Akt/GSK-3β pathway [19]. Furthermore, GSK-3β genetic variants play a role in the therapeutic response of SSRIs in depression [20]. A 5HT_1A_R agonist, 8-hydroxy-2-(din-propylamino) tetralin (8-OH-DPAT), was found to inactivate GSK-3β and the PI3K/Akt pathway was involved in this process[18, 21]. Therefore, we hypothesized that the 5-HT_1A_R mediated PI3K/Akt/GSK-3β pathway is responsible for reduced tau hyperphosphorylation in SSRIs-treated primary hippocampal neurons.

To this purpose, we treated the primary hippocampal neurons with Aβ_1-42_ to induce tau hyperphosphorylation, and then we examined whether escitalopram could attenuate tau hyperphosphorylation. Subsequently, we investigated whether the 5-HT_1A_R mediated PI3K/Akt/GSK-3β pathway was involved.

## RESULTS

### Escitalopram attenuates Aβ_1-42_-induced tau hyperphosphorylation in hippocampal neurons

**Figure 1:**
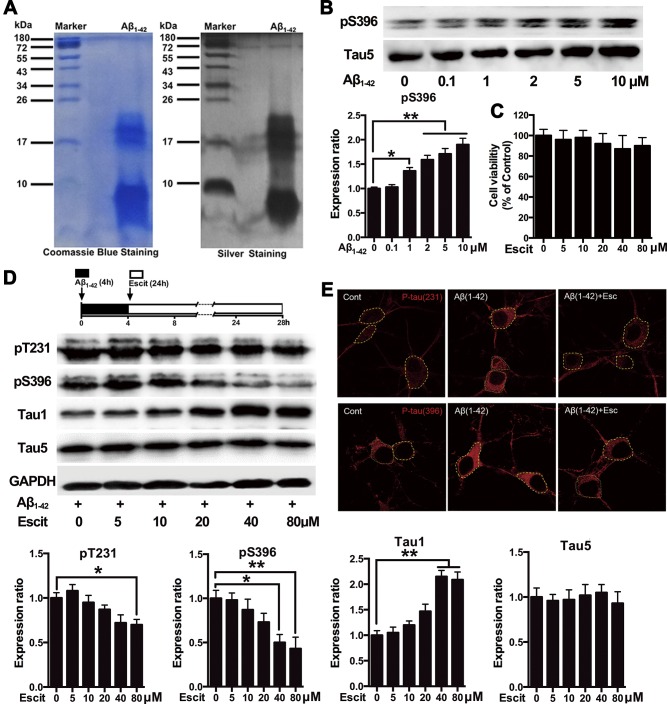
Escitalopram attenuates Aβ_**1-42**_-induced tau hyperphosphorylation in hippocampal neurons **A.** Representative coomassie blue and silver staining of the Aβ_1-42_ solution used in the cultured hippocampal neurons. **B.** Immunoblots of tau phosphorylated at pS396 site in the cultured hippocampal neurons incubated with Aβ_1-42_ for 4 h at the concentrations indicated. Tau5 was used for normalization. Data were expressed as means ± SEM (*n* = 3; *p < 0.05, **p < 0.01). B and **C.** Cells were treated with Aβ_1-42_ (2 μM) for 4 h, and then incubated with escitalopram at the concentrations indicated for 24 h in fresh medium. Cell viability was detected by the MTT assay (C). Immunoblots of tau phosphorylated at pT231, pS396, Tau1 and Tau5 **D.** Tau5 or GAPDH was used for normalization. Data were means ± SEM (*n* = 3; *p < 0.05, **p < 0.01). **E.** Representative of p-Tau (Thr231) and p-Tau (Ser396) immunofluorescence from the cultured hippocampal neurons incubated with 80μM escitalopram for 24 h in the presence of pretreatment with Aβ_1-42_ (2 μM) for 4h. p-Tau (Thr231) and p-Tau (Ser396) were labeled with red. Similar results were observed in each of three experiments. Scale bar, 10μm. Escit, Escitalopram.

As revealed by coomassie blue and silver staining, the Aβ_1-42_ preparation was almost exclusively composed of low-molecular-weight Aβ_1-42_ oligomers (Figure 1A). It produced one band likely to represent Aβ monomer (molecular weight 4.5 kDa) and two evident bands probably representing Aβ trimers and tetramers (molecular weight ∼17 kDa). Western blotting results indicated that Aβ_1-42_ at concentrations higher than 1μM significantly increased tau phosphorylation at pS396 site in primary hippocampal neuron cultures (Figure 1B). Since previous reports suggested that Aβ_1-42_ at higher concentrations induced neurotoxicity [22], 2 μM Aβ_1-42_ was considered optimum to induce tau hyperphosphorylation in our study. The MTT assay showed that escitalopram did not affect the neuronal viability at concentrations from 5 to 80 μM (Figure 1C). As shown in Figure 1D, escitalopram decreased Aβ_1-42_-induced tau phosphorylation in a concentration-dependent manner, while it had no effect on Tau5 that represents the total tau protein. Escitalopram (80 μM) significantly decreased tau phosphorylation at Thr231 and Ser396, while increased Tau1 that indicates the unphosphorylated tau protein. Immunofluorescence results also showed that Aβ_1-42_ treatment increased the tau phosphorylation, while escitalopram (80 μM) attenuated the tau phosphorylation (Figure 1E).

**Figure 2:**
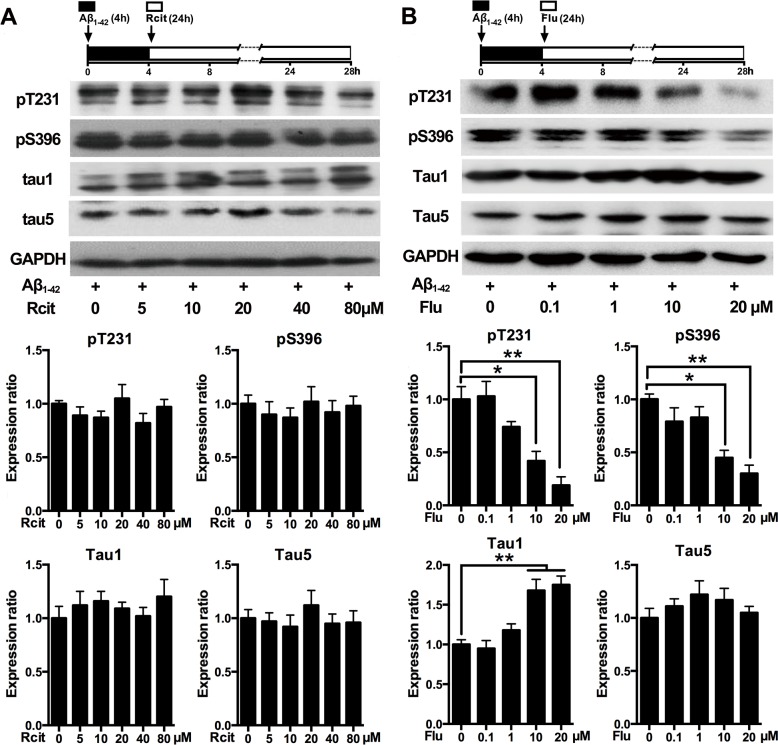
Effects of R-citalopram and fluoxetine on Aβ_**1-42**_-induced tau hyperphosphorylation in hippocampal neurons Immunoblots of tau phosphorylated at pT231, pS396, Tau1 and Tau5 in the cultured hippocampal neurons. Cells were treated with Aβ_1-42_ (2 μM) for 4 h, and then incubated with R-citalopram **A.** or fluoxetine **B.** at the concentrations indicated for 24 h in fresh medium. Tau5 or GAPDH was used for normalization. Data were represented as means ± SEM (*n* = 3; *p < 0.05, **p < 0.01). Rcit, R-citalopram; Flu, fluoxetine.

To determine whether the decreased tau phosphorylation was due to the pharmacological action of escitalopram, its enantiomer, R-citalopram, which is relatively much less active as an SSRI [23], was used. The western blotting results showed that different doses of R-citalopram had no effect on Aβ_1-42_-induced tau hyperphosphorylation at Thr231, Ser396 and Tau-1 epitopes (Figure 2A). To investigate whether the decreased tau phosphorylation was unique to escitalopram or for the SSRIs group, another SSRI, fluoxetine was used. As shown in Figure 2B, fluoxetine also decreased Aβ_1-42_-induced tau hyperphosphorylation in a concentration-dependent manner. Fluoxetine at 20 μM significantly decreased tau phosphorylation at Thr231 and Ser396, and increased Tau1, while it had no effect on Tau5.

### Activation of PI3K/Akt/GSK-3β pathway contributes to the anti-hyperphosphorylation role of escitalopram

**Figure 3:**
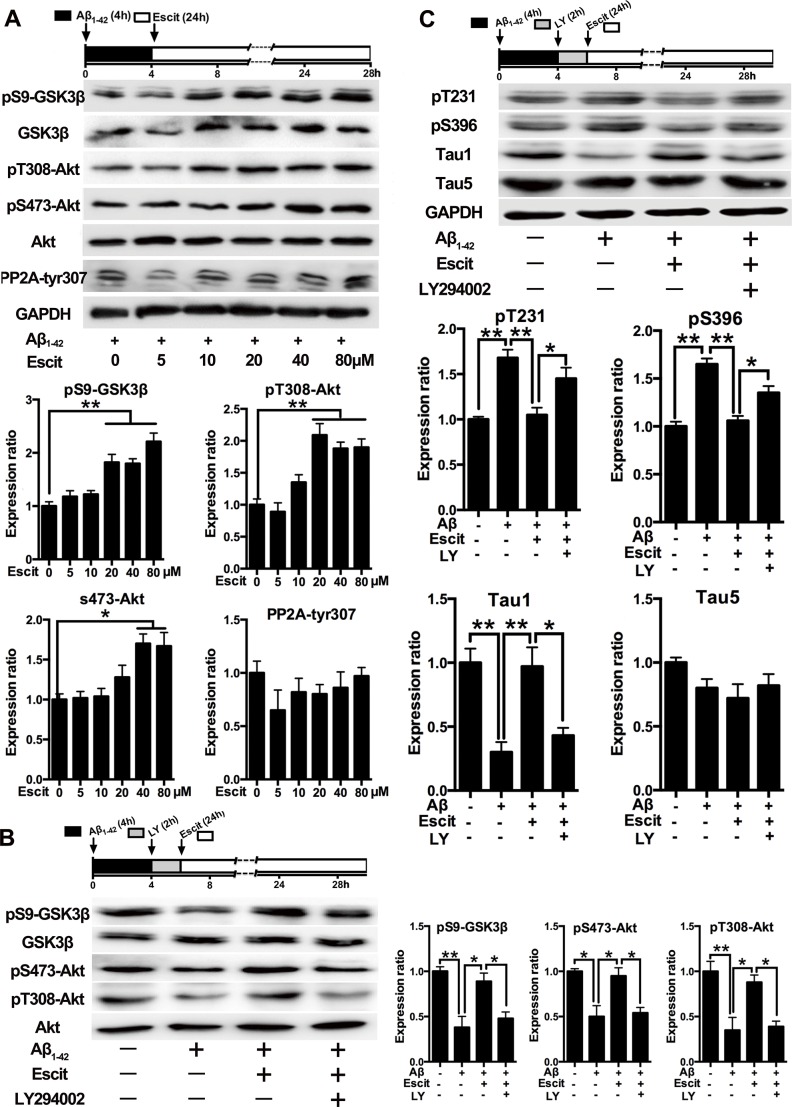
Activation of PI3K/Akt/GSK-3β pathway contributes to the anti-hyperphosphorylation role of escitalopram **A.** Immunoblots of pS9-GSK-3β, total GSK-3β, pT308-Akt, pS473-Akt, total Akt, p-PP2Ac (tyr307) in the cultured hippocampal neurons. Cells were treated with Aβ_1-42_ (2 μM) for 4 h, and then incubated with escitalopram at the concentrations indicated for 24 h in fresh medium. The respective total protein or GAPDH was used for normalization. Data were expressed as means ± SEM (*n* = 3; *p < 0.05, **p < 0.01). **B.** and **C.** Immunoblots of pS9-GSK-3β, total GSK-3β, pT308-Akt, pS473-Akt, total Akt (B) and tau phosphorylated at pT231, pS396, Tau1 and Tau5 (C) in the cultured hippocampal neurons. Cells were treated with Aβ_1-42_ (2 μM) for 4 h, and then incubated with escitalopram (80 μM) for 24 h in fresh medium with or without the pretreatment with LY294002 (10 μM, 2 h). Tau5 or GAPDH was used for normalization. Data were expressed as means ± SEM (*n* = 3; *p < 0.05, **p < 0.01). Escit, escitalopram; LY, LY294002.

As GSK-3β is the crucial kinase for tau hyperphosphorylation and phosphatase 2A (PP2A) is the key phosphatase in tau dephosphorylation, the activities of GSK-3β and PP2A were measured. As shown in Figure 3A, escitalopram increased the level of pS9-GSK-3β (inactivated form) in a concentration-dependent manner, while it had no significant effect on the level of pY307-PP2A_C_ (inactivated form). Furthermore, the phosphorylation of Akt, a critical upstream regulator of GSK-3β, was dose-dependently increased by escitalopram. In addition, LY294002, a specific inhibitor of PI3K, was found to block the phosphorylation of GSK-3β (Ser9) and Akt (Ser473 and Thr308) induced by escitalopram (80 μM) (Figure 3B). Accordingly, the attenuation of tau hyperphosphorylation at pT231, pS396 and Tau1 epitopes induced by escitalopram was reversed by LY294002 (Figure 3C). Thus, these results indicate that the PI3K/Akt/GSK-3β pathway may underlie the anti-hyperphosphorylation effect of escitalopram.

### Effects of escitalopram on the PI3K/Akt/GSK-3β signaling pathway depends on 5-HT_1A_R

**Figure 4:**
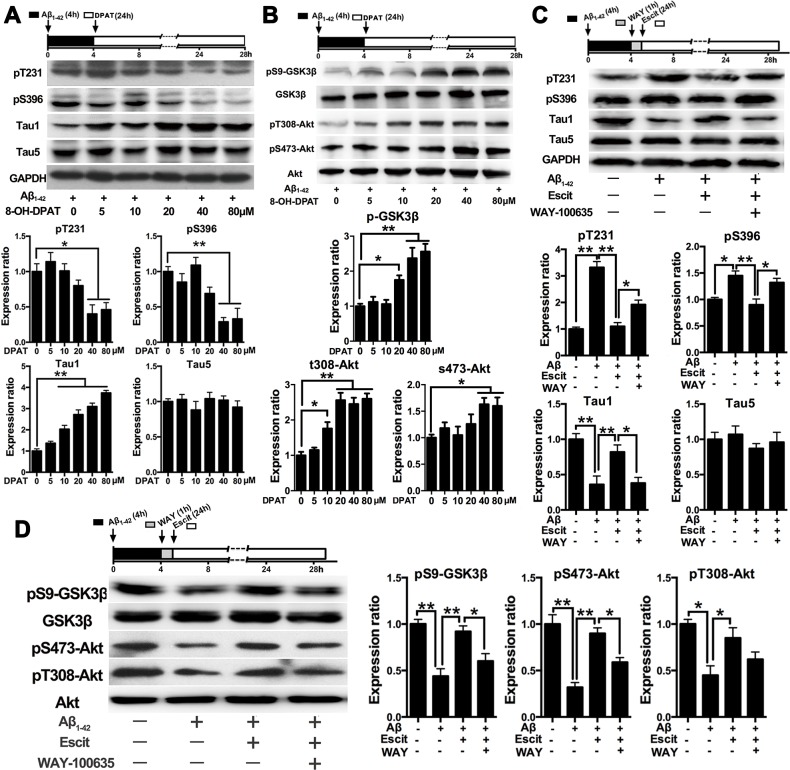
Effects of escitalopram on the PI3K/Akt/GSK-3β signaling pathway depends on 5-HT_**1A**_R **A.** and **B.** Immunoblots of tau phosphorylated at pT231, pS396, Tau1 and Tau5 (A) and pS9-GSK-3β, total GSK-3β, pT308-Akt, pS473-Akt, total Akt (B) in the cultured hippocampal neurons. Cells were treated with Aβ_1-42_ (2 μM) for 4 h, and then incubated with 8-OH-DPAT at the concentrations indicated for 24 h in fresh medium. The respective total protein or GAPDH was used for normalization. Data were represented as means ± SEM (*n* = 3; *p < 0.05, **p < 0.01). **C.** and **D.** Immunoblots of tau phosphorylated at pT231, pS396, Tau1 and Tau5 (C) and pS9-GSK-3β, total GSK-3β, pT308-Akt, pS473-Akt, total Akt (D) in the cultured hippocampal neurons. Cells were treated with Aβ_1-42_ (2 μM) for 4 h, and then incubated with escitalopram (80 μM) for 24 h in fresh medium with or without the pretreatment with WAY-100635 (10 μM, 1 h). The respective total protein or GAPDH was used for normalization. Data were expressed as means ± SEM (*n* = 3; *p < 0.05, **p < 0.01). **E.** Immunoblots of pS9-GSK-3β, total GSK-3β, pT308-Akt, pS473-Akt, total Akt in the cultured hippocampal neurons. Cells were treated with Aβ1-42 (2 μM) for 4 h, and then incubated with escitalopram (80 μM) alone or escitalopram combined with 8-OH-DPAT (80 μM) for 24 h in fresh medium with or without the pretreatment with WAY-100635 (10 μM, 1 h). The respective total protein or GAPDH was used for normalization. Data were expressed as means ± SEM (*n* = 3; *p < 0.05, **p < 0.01). DPAT, 8-OH-DPAT; Escit, escitalopram; WAY, WAY-100635.

Since 5-HT_1A_R is a critical component in the mechanism of action of SSRIs, we next examined whether 5-HT_1A_ is involved in the activation of PI3K/Akt/GSK-3β pathway induced by escitalopram. Similar to escitalopram, the 5-HT_1A_R agonist 8-OH-DPAT also decreased Aβ_1-42_-induced tau hyperphosphorylation at Thr231, Ser396 and Tau-1 epitopes in a concentration-dependent manner (Figure 4A). Furthermore, 8-OH-DPAT increased the level of pS9-GSK-3β, pT308-Akt and pS473-Akt in a concentration-dependent manner (Figure 4B). On the other hand, the protective effects of escitalopram on Aβ_1-42_-induced tau hyperphosphorylation at pT231, pS396 and Tau1 epitopes induced by escitalopram were blocked by WAY-100635, a selective antagonist of 5-HT_1A_R (Figure 4C). Additionally, the stimulatory effects of escitalopram on phosphorylation of both Ser9 on GSK-3β and Ser473 on Akt were significantly blocked by WAY-100635 (Figure 4D).

### Escitalopram improves Aβ_1-42_ induced impairment of dendritic outgrowth

**Figure 5:**
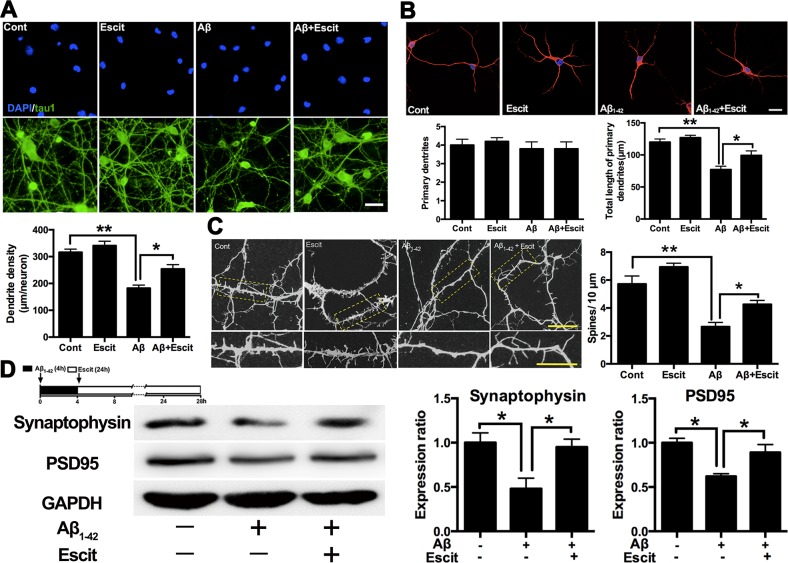
Escitalopram improves Aβ_**1-42**_ induced impairment of dendritic outgrowth **A.** Dendrite density of the neuron was detected by immunofluorescence assay. Tau 1 was labeled as green and DAPI was blue. Data were represented as means ± SEM (*n* = 3; **p < 0.01). Scale bar, 10 μm. **B.** Number of primary dendrites and total length of primary dendrites were examined by immunofluorescence assay. Tau 5 was labeled as red and DAPI was blue. Data were represented as means ± SEM (*n* = 3; *p < 0.05, **p < 0.01). Scale bar, 5 μm. **C.** Spine density was detected by immunofluorescence assay labeled with Tau 5. Data were represented as means ± SEM (*n* = 3; *p < 0.05, **p < 0.01). Scale bar, 5 μm. **D.** Immunoblots of synaptophysin and PSD95 in the cultured hippocampal neurons. GAPDH was used for normalization. Data were represented as means ± SEM (*n* = 3; *p < 0.05). Escit, escitalopram.

A dendritic outgrowth assay was performed to investigate whether escitalopram can regulate dendrite and spine morphology in hippocampal neurons. As shown in Figure 5A and 5B, escitalopram alone had no significant effect on dendritic outgrowth and spine density in hippocampal neurons under control conditions. Aβ_1-42_ treatment decreased the dendrite density and the total length of primary dendrites, while escitalopram treatment up-regulated the dendrite density and the total length of primary dendrites, neither Aβ_1-42_ nor escitalopram had an effect on the number of primary dendrites. Statistical analyses showed that Aβ_1-42_ treatment significantly decreased the density of spines, while escitalopram treatment reversed this significantly (Figure 5C). Western blotting results showed that Aβ_1-42_ treatment decreased levels of synaptophysin and PSD95, and again escitalopram treatment significantly reversed these effects (Figure 5D).

## DISCUSSION

The present study revealed that escitalopram could protect cultured hippocampal neurons against Aβ_1-42_-induced tau hyperphosphorylation through the PI3K/Akt/GSK-3β pathway, with the involvement of 5-HT_1A_R. Furthermore, escitalopram may have a potent effect on neurite outgrowth of hippocampal neurons exposed to Aβ_1-42_.

SSRIs are widely used in the treatment of depression. Interestingly, recent studies have found that SSRIs reduce the risk of AD in depressed individuals [24] and have a positive role in hindering the progression of AD and improving patients' daily performance [8, 9, 25]. In preclinical studies, a favorable cognitive-improving effect of SSRIs has been proved [10, 11, 26]. Citalopram, paroxetine and fluoxetine have been found to modulate the processing of amyloid precursor protein *in vitro* [27] and to lower Aβ and plaque densities *in vivo* [13, 26, 28]. Furthermore, it was reported that paroxetine reduced tau immunoreactive hippocampus CA1 neurons in 3xTg AD mice [26]. Our previous study has also revealed that escitalopram ameliorated forskolin-induced tau hyperphosphorylation in HEK293/tau441 cells [14]. Here we found that both escitalopram and fluoxetine attenuated Aβ_1-42_-induced tau hyperphosphorylation in primary hippocampal neurons, which further demonstrated that SSRIs could lessen tau pathology.

The mechanism by which SSRIs inhibit tau hyperphosphorylation is unknown. Akt/GSK-3β is the most implicated signaling pathway in regulating tau phosphorylation [29]. It was demonstrated that stimulation of GSK-3β both *in vitro* and *in vivo* induces tau hyperphosphorylation with impairments of the cognitive functions, whereas inhibition of GSK-3β improves tau pathologies and memory deficit [30]. In addition, several recent publications have implicated the Akt/GSK-3β pathway as the mechanism of action of some SSRIs antidepressants. For example, the activation effects of escitalopram, paroxetine, sertraline and fluoxetine on Akt have been previously reported in hippocampal neuron cultures, neural stem cells, and rat brain [31, 32]; the inhibition effects of fluoxetine on GSK-3β was also reported in mice brain and cultured neural precursor cells [16, 33]. Our present study showed that Akt was activated and GSK-3β was inhibited following escitalopram administration, while pharmacological inhibition of PI3K abolished the effect of escitaloram on tau phosphorylation, in agreement with previous results and suggesting that the neuroprotective effect of escitalopram on Aβ_1-42_-induced tau hyperphosphorylation is directly related to the activation of PI3K/Akt/GSK-3β signaling pathway.

The underlying mechanism of GSK-3β inhibition induced by escitalopram is also unknown. The primary action of SSRIs is based on the inhibition of serotonin reuptake to elevate synaptic 5-HT concentrations, thereby activating postsynaptic 5-HT receptors and triggering downstream intracellular signaling cascades. Among different serotonin receptors, the 5-HT_1A_R has been most implicated in mood and cognition. The density of 5-HT_1A_R is diminished in the brain in AD patients prior to the appearance of clinical symptoms [5]. Activation of the 5-HT_1A_R is a critical component in the action mechanism of SSRIs [34]. Increasing evidence indicates that the PI3K/Akt /GSK-3β pathway can be regulated by 5-HT_1A_R. Selective agonists for 5-HT_1A_R stimulated an activation of Akt [35] and an inhibition of GSK-3β [18] *in vitro* and *in vivo*. Furthermore, a follow-up study revealed that the regulation of PI3K/Akt/GSK-3β by 5-HT_1A_R and fluoxetine is an important signaling mechanism for serotonin-regulated behaviors [21]. Therefore, we speculate that 5-HT_1A_R may play an important role in escitalopram-induced activation of the Akt/GSK-3β pathway. In the present study, we found that the 5-HT_1A_R agonist 8-OH-DPAT activated the Akt/GSK-3β pathway, besides, the up-regulation of GSK-3β and Akt phosphorylation induced by escitalopram was blocked by the 5-HT_1A_R antagonist WAY-100635, providing powerful evidence to support our speculation.

Several previous studies have clearly pointed out the neuroprotective and neurotrophic potential of 5-HT_1A_R agonists [36, 37], however, few researches have investigated whether 5-HT_1A_R is directly relate to tau phosphorylation. In the present study, we found that the 5-HT_1A_R agonist 8-OH-DPAT significantly decreased Aβ_1-42_-induced tau hyperphosphorylation, while the 5-HT_1A_R antagonist WAY-100635 reversed the attenuation effects of escitalopram on tau hyperphosphorylation. Here we provided new evidence that a 5-HT_1A_R agonist decreased tau hyperphosphorylation to further support the potential of 5-HT_1A_ receptor agonists as neuroprotectants. More importantly, these findings firmly demonstrated that 5-HT_1A_R is a key molecule involved in the attenuation of tau hyperphosphorylation by escitalopram.

Previous research has indicated that abnormal hyperphosphorylation of tau protein contributes to disturbance of neural plasticity in AD [38-40]. Therefore, we subsequently investigated whether escitalopram enhanced dendritic outgrowth in hippocampal neurons. We observed that escitalopram significantly enhanced dendritic outgrowth and increased dendritic spines in hippocampal neuron cultures exposed to Aβ_1-42_. Our previous study has reported that escitalopram rescued synaptic deficits in depressive-like rats [41]. Moreover, several studies have revealed that the disturbance of synaptic plasticity recovered when tau hyperphosphorylation was reversed pharmacologically or with genetic technology [39, 40], consistent with our results. However, whether the above-mentioned PI3K/Akt/GSK-3β pathway is also related to the enhancement of synaptic plasticity induced by escitalopram has yet to be determined. The PI3K/Akt/GSK-3β pathway is involved in long-term potentiation [42], neurite outgrowth [43], axonal outgrowth and dendritic plasticity [44] *in vitro* and *in vivo*. Moreover, a PI3K inhibitor significantly reduced the promoting effects of antidepressant drugs on dendritic outgrowth in hippocampal neurons [31]. Thus, we presume that the PI3K/Akt/GSK-3β pathway is also related to the improvement of dendritic outgrowth induced by escitalopram. However, further work is needed to fully define the mechanisms.

We acknowledge that the doses of each SSRI administered in our experiment were higher than those normally found in brain tissue, however, such high doses are routinely used in other *in vitro* studies [31, 45]. Moreover, we cannot draw conclusions about *in vivo* effects of escitalopram on tau hyperphosphorylation from our *in vitro* data; further studies are also needed to determine if the findings generalize to all SSRIs or even other antidepressant drugs.

In conclusion, we demonstrated that escitalopram attenuates tau hyperphosphorylation *via* the PI3K/Akt/GSK-3β signaling pathway that links 5-HT_1A_R activation. Our findings shed new light on the neuroprotective effect of escitalopram involved in tau hyperphosphorylation and support a role for 5-HT_1A_R mediated Akt/GSK-3β pathway in tau phosphorylation. Finally, these may provide theoretical evidence supporting the potential of escitalopram in the treatment of tau hyperphosphorylation associated disease, including AD.

## MATERIALS AND METHODS

### Drugs and reagents

Aβ protein fragment 1-42 (Aβ_1-42_), Fluoxetine, 8-OH-DPAT, WAY-100635, LY294002 were purchased from Sigma-Aldrich (MO, USA). Escitalopram was kindly provided by H. Lundbeck A/S. Copenhagen-Valby, Denmark. R-citalopram was purchased from Santa Cruz Biotechnology (CA, USA). Escitalopram, fluoxetine, R-citalopram, 8-OH-DPAT, WAY-100635 and LY294002 were dissolved in DMSO, then were diluted using cell culture medium without bovine serum with the final concentration of DMSO less than 0.05%.

### Aβ_1-42_ preparation

Aβ_1-42_ was dissolved in DMSO, and incubated for 24 h at 37°C to allow for fibril formation [22]. In order to examine the extent and type of Aβ_1-42_ fibrils formed, the Aβ_1-42_ preparations (20 μg) were separated by electrophoresis on a 16.5% tris-tricine gel, and then the gel was visualized by coomassie brilliant blue R-250 staining (Beyotime, Haimen, China) or silver staining using a Fast Silver Stain Kit (Beyotime).

### Primary hippocampal neuron cultures

Primary cultures of hippocampal neurons were prepared from fetal brains (embryonic day 18; E18) obtained from female Sprague-Dawley rats (Experimental Animal Center of Southeast University). All studies involving animals were conducted in accordance with the National Institutes of Health guide for the care and use of Laboratory animals. Animal procedures undertaken were approved by Jiangsu Animal Care and Use Committee and every effort was made to minimize animal suffering. Briefly, the brains were exposed, and then the hippocampal tissues were dissociated in HBSS (Invitrogen, NY, USA) containing 0.125% trypsin solution (Gibco, NY, USA) for 15 min at 37°C. Subsequently, the digestion was terminated with DMEM (Gibco) containing 10% fetal bovine serum (Gibco). Finally, the dispersed tissues were centrifuged at 2000 rpm for 5 min and were resuspended in Neurobasal medium (Invitrogen) containing 2% B27 supplement (Gibco), 0.5mM L-glutamine (Gibco), 20 IU/ml penicillin and 20 IU/ml streptomycin. For the Western blotting procedure, neurons were plated onto six-well plates coated with poly-D-lysine (100μg/ml; Sigma-Aldrich) at a density of 2×10^6^ per well. For the immunofluorescence staining procedure, neurons were plated in cover slips at a density of 2×10^4^ cells/cm^2^. Cell cultures were kept in a humidified incubator containing 95% air and 5% CO_2_ at 37°C. The culture medium was replaced with fresh Neurobasal/B27 medium every 2-3 days. The purity of the neurons used in experiments was about 95%. The cultures were maintained for 14 days before being harvested for further analysis.

### Cell viability assay

Cell viability was determined using the 3-(4,5-dimethyl-2-thiazolyl)-2,5-diphenyl-2-H-tetrazolium bromide (MTT) assay. After treatment, primary hippocampal neurons were treated with 0.5 mg/ml MTT for 4 h at 37°C. The formazan crystals were dissolved in 100μl of DMSO and the absorbance was measured at 570 nm in a microplate reader (Multiskan GO, Thermo Scientific, NY, USA). Cell survival rates were expressed as percentages of the control group.

### Western blotting

Primary hippocampal neuron cultures were collected, washed twice with ice-cold phosphate-buffered saline (PBS) and solubilized in ice-cold lysis buffer (Beyotime) containing protease inhibitor (Roche, Laval, Quebec, Canada). The cell lysates were centrifuged at 12000g for 15 min at 4°C. The BCA kit (Pierce, Thermo Scientific, NY, USA) was used to detect the protein concentration. The samples containing equivalent amounts of protein (20 μg) were separated by SDS-PAGE and transferred to PVDF membranes (Merck Millipore, Darmstadt, Germany). The blots were blocked by 5% nonfat milk for 1h at room temperature, and then the membranes were incubated with the following primary antibodies diluted in blocking solution at 4°C overnight: mouse monoclonal Tau5 (1:5000; BioSource, NY, USA), mouse monoclonal Tau1 (1:5000; Merck Millipore),rabbit polyclonal anti-pTau (Thr231) (1:2000; Invitrogen), rabbit polyclonal anti-pTau (Ser396) (1:2000; Invitrogen), mouse monoclonal anti-GSK-3β (1:1000; Cell Signaling, MA, USA), rabbit monoclonal anti-pGSK-3β (Ser9) (1:1000; Cell Signaling), rabbit monoclonal anti-Akt (1:1000; Cell Signaling), rabbit monoclonal anti-pAkt (Ser473) (1:1000; Cell Signaling), rabbit polyclonal anti-pAkt (Thr308) (1:500; Bioworld, MN, USA), rabbit polyclonal anti-pPP2Ac (Tyr307) (1:500; Santa Cruz), rabbit monoclonal anti-PSD95 (1:2000; Abcam, MA, USA) and rabbit monoclonal anti-synaptophysin (1:1000; Merck Millipore). Internal control was performed using GAPDH antibody (1:5000; Sigma-Aldrich). After washing with TBST buffer for three times, the membranes were incubated for 1h with horseradish peroxidase-conjugated secondary antibody (goat anti- rabbit IgG, goat anti-mouse IgG) (1:5000; Invitrogen). The membranes were then processed with ECL Western blotting reagents (Pierce), and then were detected using Image Quant LAS 4000 mini system (GE Healthcare, Japan). The sum optical density was quantitatively analyzed by Quantity One software (Bio-Rad, Richmond, CA, USA).

### Immunofluorescence staining

Cells were washed three times in PBS and fixed with 4% paraformaldehyde at room temperature for 20 min. Then, cells were treated with 0.3% Triton X-100 for 5 min on ice. After washing, cells were blocked with 5% BSA for 30 min at room temperature and then incubated with rabbit polyclonal anti-pTau (Thr231) (1:400), rabbit polyclonal anti-pTau (Ser396) (1:400), mouse monoclonal Tau1 antibody (1:500) or mouse monoclonal Tau5 antibody (1:500) at 4°C overnight. Cells were washed three times in PBST and incubated with Alexa Fluor 488 or Alexa Fluor 594 goat anti-rabbit, goat anti-mouse secondary antibody (1:2000; Invitrogen) for 1h. Finally, the cells were rinsed with PBST, stained with DAPI (Beyotime) and observed under Olympus FV 1000 Viewer (Olympus, Tokyo, Japan). For the morphological analysis of dendrites/spines, five fields were randomly selected from each sample and three independent experiments for each sample were performed. The images were captured by a person blind to their identities and were analyzed using Image J software 1.48 (NIH, Bethesda, USA).

### Statistical analysis

Data were presented as mean ± standard error of the mean (SEM). One-way analysis of variance (ANOVA) followed by Tukey post hoc test were used to compare the differences between means in more than two groups by GraphPad Prism 6.01. A probability value of *P* < 0.05 was considered to be statistically significant.
